# Improving architectural traits of maize inflorescences

**DOI:** 10.1007/s11032-021-01212-5

**Published:** 2021-02-27

**Authors:** Zongliang Chen, Andrea Gallavotti

**Affiliations:** 1 Waksman Institute of Microbiology, Rutgers University, Piscataway, NJ 08854-8020 USA; 2 Department of Plant Biology, Rutgers University, New Brunswick, NJ 08901 USA

**Keywords:** Maize, Inflorescence meristem, CLAVATA-WUSCHEL, Phytohormones, Transcription factors

## Abstract

The domestication and improvement of maize resulted in radical changes in shoot architecture relative to its wild progenitor teosinte. In particular, critical modifications involved a reduction of branching and an increase in inflorescence size to meet the needs for human consumption and modern agricultural practices. Maize is a major contributor to global agricultural production by providing large and inexpensive quantities of food, animal feed, and ethanol. Maize is also a classic system for studying the genetic regulation of inflorescence formation and its enlarged female inflorescences directly influence seed production and yield. Studies on the molecular and genetic networks regulating meristem proliferation and maintenance, including receptor-ligand interactions, transcription factor regulation, and hormonal control, provide important insights into maize inflorescence development and reveal potential avenues for the targeted modification of specific architectural traits. In this review, we summarize recent findings on the molecular mechanisms controlling inflorescence formation and discuss how this knowledge can be applied to improve maize productivity in the face of present and future environmental challenges.

## Introduction

**Fig. 1:**
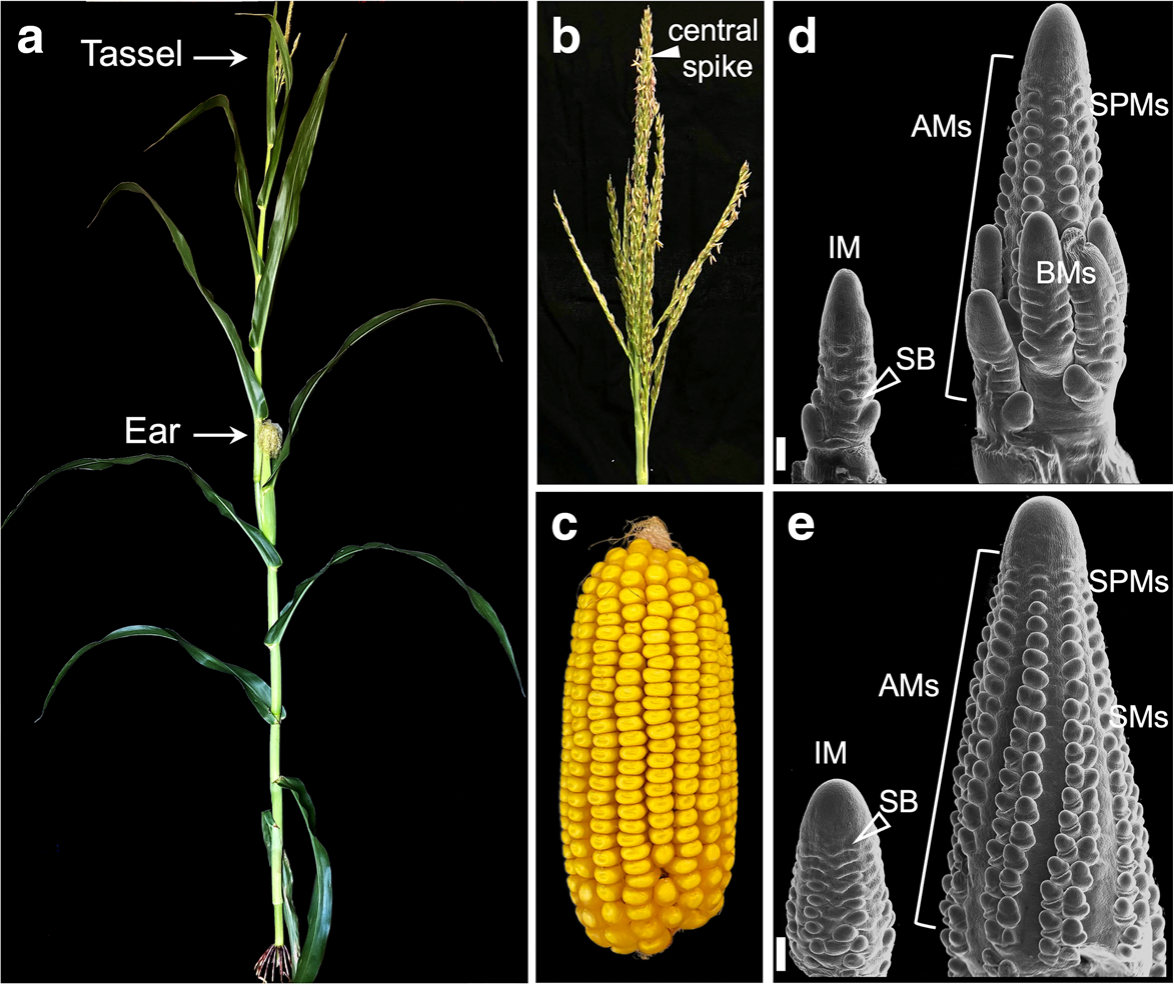
Maize inflorescence architecture. **a** Mature maize plant with tassel and ear. **b** Mature tassel. **c** Mature ear. **d**, **e** Scanning electron microscope (SEM) images of developing tassel (**d**) and ear primordia (**e**) in early and later stages of development. IM, inflorescence meristem; SB, suppressed bract; BMs, branch meristems; AMs, axillary meristems; SPMs, spikelet-pair meristems; SMs, spikelet meristems. Scale bars: 200 μm

As a monoecious plant, maize male and female reproductive organs are borne on physically separated inflorescences that are phenotypically distinguishable and exhibit distinct architectures (Fig. 1a). The tassel is the male staminate inflorescence located at the top of the plant and is composed of a central spike and several long branches borne at the bottom of the spike (Fig. 1b). The female pistillate inflorescences, commonly known as ears, originate from axillary buds formed at the stem nodes and lack long branches (Fig. 1c). Both tassel and ear initiate as bisexual inflorescences with a remarkably similar architecture at the early stages of development. However, during the development to mature structures, several distinct changes occur in both inflorescences, among which are gynoecium abortion in tassels and stamens abortion in ear shoots, leading to distinct unisexual flowers contained in spikelets (Cheng et al. 1983; Vollbrecht and Schmidt 2009).

The architecture of maize inflorescences and in particular the size of ears and the number of kernel rows on each ear are crucial traits directly related to yield (Li et al. 2018). Maize tassel architecture, including the length and number of branches, also influences yield although since pollen production is typically not a limiting factor (Westgate et al. 2003), most breeding efforts have selected for decreased tassel size, allowing for increased light perception and the shifting of plant resources into grain production (Xu et al. 2017). Indeed, detasseled maize plants or plants with small tassels show increased yield (Hunter et al. 1969; Fischer et al. 1987), and small tassels with upright branches normally ensure sufficient pollen availability. Considerable interest has instead centered on increasing the size of maize ears that have already been drastically modified from those of the progenitor teosinte (Doebley 2004). Ear size (determined by both ear length and diameter) directly impacts grain yield by determining the number and size of seeds.

Both ear and tassel originate from inflorescence meristems (IMs) that contain small groups of pluripotent stem cells at the apical domain (Fig. 1d, e). These stem cells maintain themselves as undifferentiated cells as well as differentiate to produce new lateral organs (Somssich et al. 2016; Kitagawa and Jackson 2019). During the transition to reproductive development, the shoot apical meristem (SAM) converts into the tassel IM. Soon after establishment, the IM produces lateral primordia called suppressed bracts which subtend indeterminate axillary meristems (AMs) called branch meristems (BMs) at the base, and additional determinate AMs linearly arrayed in multiple rows (Fig. 1d; BMs are eventually responsible for the formation of long branches observed in mature tassels). Ear primordia, on the other hand, develop in the axils of leaves from vegetative axillary buds that give rise to short branches topped by IMs, and unlike in tassels, ear IMs only generate rows of determinate AMs (Fig. 1e). These determinate AMs include spikelet-pair meristems (SPMs), which in turn form spikelet meristems (SMs) that eventually give rise to floral meristems (FMs). These distinct AMs are linearly arranged on the flanks of both tassel and ear IMs, and the acquisition of different identities combined with sexual determination during floral development results in the unique male and female inflorescence architectures (Vollbrecht and Schmidt 2009). In particular, pairing of SMs in ears determines the even number of kernels typically observed in mature cobs. In essence, the meristematic activity of IMs and AMs is key to shape the overall architecture of both inflorescences. Understanding the molecular mechanisms that underlie meristem development has the potential to help engineer novel methods to improve maize yield in different environmental conditions.

**Fig. 2:**
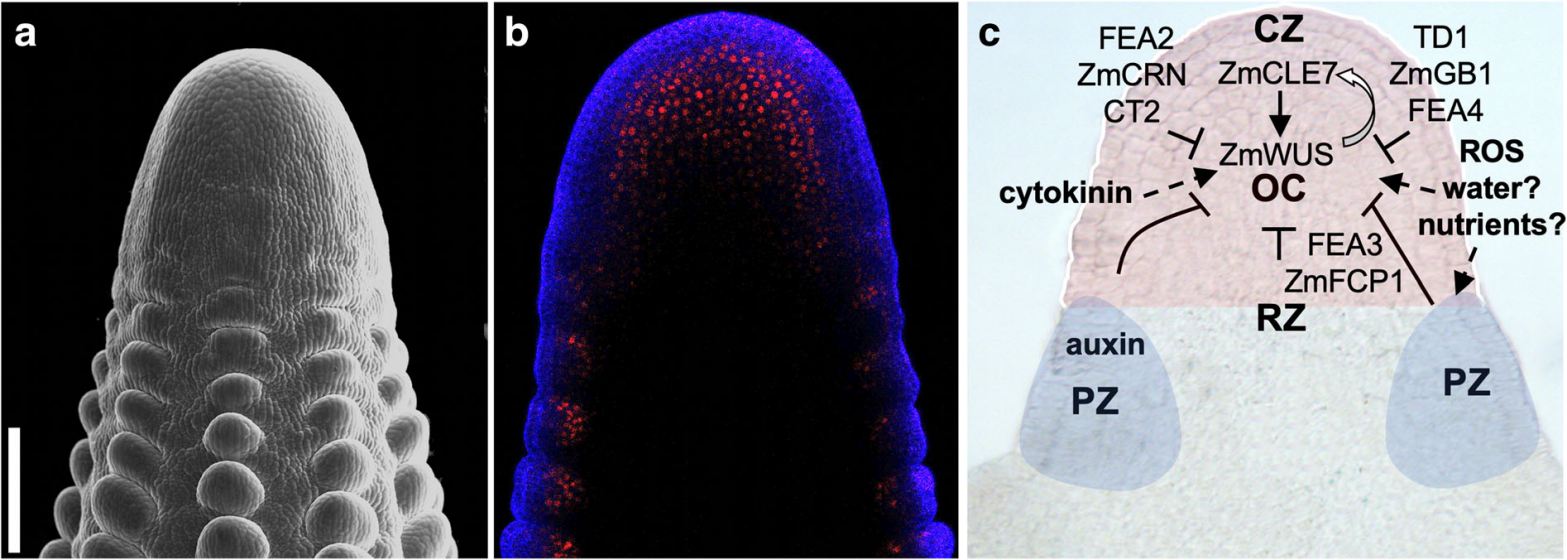
Regulation of inflorescence meristem size and function. **a** SEM of an ear inflorescence meristem. Scale bar: 200 μm. **b** Confocal microscopy image of an immature ear tip showing expression of a *pZmWUS1*::*RFP* transcriptional reporter (Je et al. 2016) in inflorescence meristems and axillary meristems. **c** Diagram of the core regulatory network and key factors controlling meristem size and function

All meristems (SAM, IMs, AMs) are highly organized structures arranged in an apical-basal pattern and serve as a reservoir of undifferentiated stem cells. Meristems contain different functional domains: (i) the central zone (CZ) maintains active pluripotent stem cells through the entire life of the plant; (ii) the peripheral zone (PZ) located at the flanks of the CZ contains cells that are the descendants of pluripotent stem cells and acquired a new identity to form lateral organ primordia; (iii) the organizing center (OC) is a small group of cells contained within the CZ and functions to control the stem cell pool size; and (iv) the rib zone (RZ) located below the organizing center gives rise to the central tissues of the stem and pushes the entire apical meristem upward (Fig. 2).

To sustain apical reservoirs of stem cells and generate lateral primordia, plants use multiple broadly conserved strategies involving receptor-ligand interactions, transcription factor networks, and hormonal regulation. An active and important area of research is aimed at understanding how these pathways coordinate their function and how environmental factors influence them. For example, to achieve maximum production, potential maize needs to be well fertilized and watered, and it is critical that nutrient and water availability sustain inflorescence growth throughout development (Hussain et al. 2019; Durbak et al. 2014; Borras and Vitantonio-Mazzini 2018). In maize, most of the key genes regulating inflorescence architecture were identified by characterizing developmental mutants with abnormal inflorescence meristems, from small meristems leading to misshapen leaves and inflorescences, to enlarged meristems with extra florets.

## The regulation of maize inflorescence meristem size and function

The functional domains of meristems (CZ, PZ, RZ, and OC) are tightly regulated to maintain meristem size and function, and the CLAVATA-WUSCHEL (CLV-WUS) negative feedback loop coordinates domain-domain interactions and balances meristem renewal and organ differentiation (Kitagawa and Jackson 2019). WUS is a homeodomain transcription factor (TF) expressed in the OC of apical meristems and is transported via plasmodesmata to the CZ to promote the proliferation of stem cells (Mayer et al. 1998; Yadav et al. 2011). In the CZ, WUS activates the *CLV3* gene, encoding a short signaling peptide perceived by a series of receptor complexes that in turn repress *WUS* expression (Mayer et al. 1998; Brand et al. 2000; Schoof et al. 2000). Activation of *CLV3* in the OC is prevented by the action of WUS in conjunction with HAIRY MERISTEMs (HAMs), GRAS-transcriptional regulators (Zhou et al. 2015; Zhou et al. 2018). While this pathway was first described in *Arabidopsis*, in this section we highlight some of the major players characterized in maize.

In *Arabidopsis*, *wus* mutants have small and unstable SAMs and genetic analysis revealed that *WUS* plays a central role in maintaining the number of pluripotent stem cells in shoot meristems (Kitagawa and Jackson 2019). The maize B73 genome encodes two duplicated *WUS* paralogs, *ZmWUS1* and *ZmWUS2* (Nardmann and Werr 2006); however, the molecular function of both *ZmWUS* genes is unknown due to the lack of characterized mutations in maize. The expression pattern of *ZmWUS1* in the presumptive organizing center of the SAM and IMs (Fig. 2b), as well as the expansion of *ZmWUS1* expression in the IMs of *fasciated ear3* (*fea3*), a mutant with enlarged ear IMs (a phenomenon called fasciation), suggests that *ZmWUS1* promotes stem cell proliferation in maize shoot meristems as well (Je et al. 2016; Nardmann and Werr 2006). Notwithstanding, single- and double-knock-out mutants of *ZmWUS* and possibly other *WOX* genes are needed to identify the role of *ZmWUS*1 and *ZmWUS2* in maize meristems. This is important because in monocots *WUS* orthologs may play a different function than that reported in *Arabidopsis*, given that the rice WUS ortholog is not required for SAM maintenance (Lu et al. 2015; Tanaka et al. 2015; Nardmann and Werr 2006; Tanaka and Hirano 2020).

Characterization of the CLV-WUS pathway in maize has been possible due to the analysis of a series of fasciated ear mutants. The *CLAVATA* genes encode CLV ligands and CLV receptors, and loss-of-function mutations in *Arabidopsis CLV1*, *CLV2*, and *CLV3* genes produce bigger meristems (Clark et al. 1997; Fletcher et al. 1999; Jeong et al. 1999). The maize *CLV1* ortholog is *THICK TASSEL DWARF1* (*TD1*), which encodes a leucine-rich repeat receptor-like kinase (LRR-RLK), while the *CLV2* ortholog is *FASCIATED EAR2* (*FEA2*) that encodes an LRR receptor-like protein (LRR-RLP). Null mutations of both *td1* and *fea2* result in overproliferating inflorescence meristems (Taguchi-Shiobara et al. 2001; Bommert et al. 2005), indicating a conserved role of CLV1 and CLV2 in the regulation of meristem size in distantly related species. While two potential maize *CLV3* orthologs exist, *ZmCLE7* and *ZmCLE14* (Je et al. 2016), *Zmcle7* null mutants were shown to have a fasciated ear phenotype (Rodriguez-Leal et al. 2019). As a secreted peptide, ZmCLE7 is perceived by FEA2, which functions in a signal transduction pathway that includes the heterotrimeric G protein α subunit COMPACT TASSEL2 (CT2) and the β subunit ZmGB1 (Je et al. 2018; Bommert et al. 2013; Wu et al. 2020). An additional independent signal transduction pathway involves the ZmFCP1 peptide and the LRR-RLP FEA3 (Je et al. 2016). ZmFCP1 is perceived by FEA3, but it can also be perceived by FEA2 together with the pseudokinase ZmCORYNE (Je et al. 2018). Altogether, these signaling pathways are believed to contribute to the positioning of *ZmWUS* expression within maize meristems (Je et al. 2016) (Fig. 2b, c).

As a seed crop, the overall yield of maize is highly dependent on the number of kernels per ear, with wider IMs generally having more room for extra AMs and taller IMs resulting in longer ears, eventually resulting in more seeds attached to each cob (Jia et al. 2020; Je et al. 2016; Bommert et al. 2013). Although mutations in most *CLV* genes in maize condition short and fasciated ears, they still have the potential to increase the number of kernel rows and eventually grain production. Indeed, it was shown that weak alleles of both *fea2* and *fea3* not only have greater IM diameter that increases rows of kernels in ears but also produce ears of comparable length to normal ears, even in hybrid combinations (Bommert et al. 2013; Je et al. 2016). Similar work in tomato has shown that CRISPR-Cas9 editing of cis-regulatory elements in the *SlCLV3* promoter region has quantitative effects on floral organ number and fruit size (Rodriguez-Leal et al. 2017).

The identification and cloning of quantitative trait loci (QTL) that underlie agronomic traits such as kernel number per row (KNR) in ears is another promising approach to uncover the molecular causes of yield-related traits. A major QTL, *KNR6*, was found to have pleiotropic effects in ear length and kernel number per row. *qKNR6* encodes an active serine/threonine-protein kinase that phosphorylates an ARF GTPase-activating protein (AGAP) which controls the activity of the GTP-binding proteins (Jia et al. 2020). Overexpression of *qKNR6* results in longer ears with a higher kernel number per row. In contrast, reduced expression of *qKNR6* mediated by RNA interference led to shorter ears with reduced numbers of kernels per row. Similarly, null mutations in the *ZmAGAP* gene generated by CRISPR-Cas9 phenocopied KNR6 silencing, suggesting that both genes are involved in controlling ear length and ultimately yield (Jia et al. 2020).

UNBRANCHED2 (UB2) and UB3 are two functionally redundant TFs that belong to the SQUAMOSA PROMOTER BINDING (SBP)-box TF family (Chuck et al. 2014). Both genes are expressed in the periphery of shoot meristems, and *ub2*;*ub3* double mutants show enlarged inflorescence meristems and increased kernel row number. While single *ub2* and *ub3* mutants have no obvious phenotype, an additional study identified a major QTL for kernel row number, *KRN4*, and revealed that a transposon insertion located ~ 60 kb downstream of *UB3* is responsible for reducing its expression and therefore increasing meristem size (Liu et al. 2015). Additionally, *UB3* is one of the downstream targets of GROWTH-REGULATING FACTOR (GRF)-INTERACTING FACTOR 1 (GIF1), for which null mutants (*gif1*) show fasciated ears similar to those of *ub2*;*ub3* double mutants (Zhang et al. 2018). Overall, increasing seed yield in maize by tinkering with the CLV-WUS pathway or related pathways is an exciting application for the basic molecular knowledge about meristem size regulation that has emerged in recent years. Promising results using a *fea2* weak alleles in field trials suggest that this or similar strategies have indeed the potential to significantly increase maize yield (Trung et al. 2020).

*KNOTTED1* (*KN1*) was the first identified gene in maize shown to play a critical role in the balance of stem cell renewal and organ initiation in all shoot meristems independent of the CLV-WUS pathway (Bolduc et al. 2012; Hay and Tsiantis 2010). *KN1* is the founding member of the class I KN1-like homeobox (KNOX) family (Hake et al. 2004) and is expressed in a broad domain encompassing the central and rib zones of meristems but is excluded from the peripheral zone. Ectopic expression of *KN1* and other *KNOX* genes (*ROUGH SHEATH1 RS1*, *GNARLEY1*, *LIGULELESS3* and *4*), usually leads to the formation of knots or flap tissues as well as different proximal-distal patterning defects in leaves (Schneeberger et al. 1995; Foster et al. 1999; Muehlbauer et al. 1999; Bauer et al. 2004). Recessive *kn1* loss-of-function mutants in certain inbred backgrounds, on the other hand, have severe phenotypes including the formation of small meristems and arrested shoot development (Vollbrecht et al. 2000). In other inbred lines such as B73, *kn1* mutants produce tassel and ear primordia with fewer AMs leading to tassels with barren patches and fewer branches, and ears that are either absent or much smaller in size with only a small number of seeds (Kerstetter et al. 1997). These phenotypes reveal a role for *KN1* in meristem maintenance and AM formation. Other *KNOX* genes play similar roles; for example, it was reported that a loss-of-function mutant of *RS1* while having no obvious phenotype enhanced the *kn1* phenotype, indicating unequal redundancy between *KNOX* genes (Bolduc et al. 2014).

The identification of KN1 transcriptional targets revealed a molecular link between KNOX function and the control of inflorescence architecture. A study on the KN1 genome-wide occupancy that combined chromatin immunoprecipitation sequencing (ChIP-seq) and RNA sequencing (RNA-seq) in immature ears of *kn1* mutants revealed that KN1 preferentially binds to many TFs and genes regulating hormone metabolism, transport and signaling pathways, and particularly auxin pathways (Bolduc et al. 2012). Auxin is a major hormone that shapes maize inflorescence architecture as it is crucial for, among other things, the formation of lateral primordia (see below). KN1 was shown to directly regulate auxin-related genes, including genes involved in auxin biosynthesis, signaling, efflux and influx transport, and response (Bolduc et al. 2012). Furthermore, KN1 may physically interact with the zinc-finger TF RAMOSA1 (RA1), and co-regulated targets of both factors are involved in meristem determinacy and maintenance (Eveland et al. 2014). RA1 is a key determinacy factor of spikelet-pair meristems and loss-of-function *ra1* mutants show a dramatic increase of long branches in both tassels and ears. The timing of *RA1* expression was shown to contribute to different branching patterns of grass inflorescences (Vollbrecht et al. 2005).

Additional transcriptional regulators specifically expressed in distinct domains of inflorescence meristems play important roles in maize inflorescence architecture. Two functionally redundant BELL1-like homeobox (BLH) TFs, BLH12 and BLH14, are required for the maintenance of axillary meristems, as well as tassel branch patterning (Tsuda et al. 2017). Both BLH12 and BLH14 interact with KN1 in vivo and their expression patterns overlap with that of *KN1* in shoot apical meristem and inflorescence meristems. Moreover, like a null mutation of *kn1*, *blh12/blh14* double mutants fail to maintain axillary meristems, suggesting that BLH12 and BLH14 are bona fide cofactors of KN1 in controlling the initiation of axillary meristems (Tsuda et al. 2017).

## Hormonal control of inflorescence architecture

Phytohormones are involved in almost every aspect of plant development and growth throughout the entire life of a plant. In meristems, contrasting activities between cytokinin and auxins in promoting stem cell proliferation and differentiation, respectively, are well documented (Gaillochet and Lohmann 2015). In particular, auxin is a crucial hormone for organogenesis and meristem establishment, whose concentration varies in different parts of the plant (Benkova et al. 2003; Wang et al. 2014b; Wang et al. 2014a). Locally, auxin concentration is determined by both metabolism and transport mechanisms. In maize, auxin biosynthesis involves at least two distinct enzymes, the YUCCA-like flavin monooxygenase SPARSE INFLORESCENCE1 (SPI1) and the tryptophan aminotransferase VANISHING TASSEL2 (VT2) (Gallavotti et al. 2008; Phillips et al. 2011). In *spi1* mutants, severe defects in both inflorescences are observed, including reduced numbers of branches and spikelets in tassels and small ears bearing few seeds. Intriguingly, both tassel and ear primordia show disorganized and slightly enlarged IMs with AMs grown over the tip (Gallavotti et al. 2008). The tassels of *vt2* mutants produce no tassel branches or functional spikelets, and the ears are very small and typically have barren patches on one or both sides (Phillips et al. 2011). As a mobile molecule, auxin flow and distribution among cells are controlled by polarly localized auxin efflux transporters and auxin influx carriers. In maize, *ZmAUX1* encodes an auxin influx carrier, and a null mutation of *Zmaux1* shows a reduced number of tassel branches and spikelets (Huang et al. 2017). In addition, the maize PINOID (PID) ortholog encoded by *BARREN INFLORESCENCE2* (*BIF2*) plays an important role in axillary meristem and lateral organ initiation (McSteen et al. 2007). As a serine/threonine-protein kinase, the *Arabidopsis* PID protein phosphorylates the auxin efflux transporter AtPIN1 and controls the subcellular localization of AtPIN proteins that direct auxin flow (Michniewicz et al. 2007). Similarly, BIF2 phosphorylates ZmPIN1a and regulates its subcellular localization during maize inflorescence development (Skirpan et al. 2009).

Auxin is perceived by nuclear co-receptor complexes including TRANSPORT INHIBITOR RESISTANT1/AUXIN SIGNALING F-BOX (TIR1/AFB) and AUXIN/INDOLE-3-ACETIC ACID (AUX/IAA) proteins. TIR1/AFB is the substrate-recognition subunit of the SKP1/CULLIN1/F-Box (SCF) E3 ubiquitin ligase complex, and auxin bound to TIR1/AFB promotes the interaction with AUX/IAA proteins, triggering the removal of the AUX/IAA by polyubiquitination of AUX/IAAs followed by degradation by the 26S proteasome. The degradation of AUX/IAAs releases AUXIN RESPONSE FACTOR (ARF) transcription factors to activate early auxin-responsive gene transcription when bound to auxin-responsive cis-regulatory elements (Weijers and Wagner 2016). Auxin signaling components involved in maize inflorescence development include BARREN INFLORESCENCE1 (BIF1) and BIF4, encoded by two *AUX*/*IAA* genes (*ZmIAA27* and *ZmIAA20*, respectively). *Bif1* and *Bif4* are semi-dominant mutants with fewer tassel branches and barren patches on tassels, as well as shortened ears bearing disorganized rows of seeds and areas devoid of seeds. In *Bif1* and *Bif4* mutants, point mutations in the degron domain stabilize BIF1 and BIF4 mutant proteins thus inhibiting expression of downstream auxin-response genes even in the presence of the hormone (Galli et al. 2015; Liu et al. 2019a). Genes downstream of auxin signaling that function in axillary meristem initiation and pattern both inflorescences include *BARREN STALK1* and *BARREN STALK2*, encoding two interacting transcriptional regulators (Galli et al. 2015; Yao et al. 2019).

The transcriptional repression on downstream target genes by AUX/IAA proteins requires the interaction between AUX/IAA proteins and the transcriptional corepressor TOPLESS (TPL), which changes local chromatin structure (Long et al. 2006; Krogan et al. 2012; Wang et al. 2013). In maize, the transcriptional corepressor RAMOSA1 ENHANCER LOCUS2 (REL2) is a member of the TPL family that interacts with AUX/IAA proteins and other transcription factors carrying DLN-type and RLFGV-type motifs (Liu et al. 2019b). The *rel2* mutant was first identified as a genetic enhancer of the classic *ra1* mutant (Gallavotti et al. 2010), and further phenotypic analysis revealed that *rel2* mutants show background-dependent pleiotropic phenotypes in both vegetative and reproductive development (Liu et al. 2019b). In particular, *rel2* shows severely upright tassel branches, enlarged inflorescence meristems, and increased kernel row number in ears of the A619 inbred background. Overall, these results suggest that REL2 plays a role in AM initiation and also controls inflorescence meristem size (Liu et al. 2019b). The importance of REL2 in the latter is also supported by a recent GWAS study that identified a SNP in *REL2* associated with an increased kernel row number (Parvathaneni et al. 2020). Whether this effect on the size of meristems is due to an effect on the auxin signaling pathway or other pathways is not yet known, although the transcription factor WUS is known to interact with TPL corepressor proteins (Kieffer et al. 2006; Causier et al. 2012; Zhang et al. 2014) and may therefore influence the core pathway for meristem maintenance. However, the pleiotropic phenotype of *rel2* mutants in most inbred backgrounds appears to limit its potential practical applications.

While auxin-related mutants affect suppressed bract and axillary meristem initiation as well as inflorescence meristem size, presumably through a feedback mechanism (Shi et al. 2018), these processes are inherently linked and it is yet unclear whether specific manipulations of auxin signaling in inflorescences may yield to practical bioengineering approaches. It is nonetheless conceivable that modulation of auxin response by engineering stability variants of AUX/IAA proteins (Ramos Baez et al. 2020) may alter the patterning of lateral organ primordia and consequently axillary meristems in inflorescence meristems, producing desirable architectural changes in maize inflorescences. Similar approaches have been indeed successfully used in *Arabidopsis*, where different mutations in IAA28 that alter auxin-driven degradation rates were shown to affect phyllotaxy (Moss et al. 2015).

In opposition to the role of auxin at the peripheral zone of meristems, cytokinins have long been known to promote stem cell proliferation in the meristem central zone and *WUS* itself is positively regulated by cytokinin in *Arabidopsis* (Wang et al. 2017; Zubo et al. 2017; Meng et al. 2017; Xie et al. 2018). In maize, mutant analysis mainly revealed defects during vegetative development. The maize *ABPHYL1* (*ABPH1*) gene encodes a type-A response regulator protein (ZmRR3) that regulates the distichous phyllotaxy of maize plants by negatively regulating cytokinin signaling (Giulini et al. 2004). In addition to phyllotactic defects, *abph1* mutants also develop bigger SAMs (Jackson and Hake 1999). These phenotypes are enhanced by gain-of-function mutations in the maize cytokinin receptor HISTIDINE KINASE1 kinase HAIRY SHEATH FRAYED (Muszynski et al. 2020). Conversely, defects in cytokinin biosynthesis led to premature SAM termination in maize (Knauer et al. 2019).

Gibberellic acid (GA) is known to promote, among other processes, stem elongation (Schwechheimer and Willige 2009). The binding of GA to its receptor GIBBERELLIN INSENSITIVE DWARF1 (GID1) triggers ubiquitin-dependent degradation of DELLA proteins, whose mutants have pleiotropic effects on plant architecture. In particular, DELLA proteins were shown to have a role in regulating IM size in *Arabidopsis*, and null mutations of all *Arabidopsis DELLA* genes result in enlarged IMs. While ChIP-seq in IMs revealed that DELLA proteins directly bind to the cell cycle inhibitor *KRP2* in the underlying rib meristem, null mutations in *KRP2* reduced the size of meristems without affecting stem growth, revealing a potential strategy to uncouple stem growth from meristem size (Serrano-Mislata et al. 2017). Further analysis of *della* mutants in barley showed a conserved function of DELLA proteins in this process (Serrano-Mislata et al. 2017), although it is not yet known whether this function is conserved in maize. However, among the direct targets of the maize transcription factor, KN1, described above, is the gibberellic acid biosynthetic gene, *GA2OX1*, which encodes an enzyme that inactivates GA (Bolduc and Hake 2009). By binding to two TGAC motifs in a cis-regulatory region on the first intron of *GA2OX1*, KN1 mediates activation of *GA2OX1* expression at the base of the SAM and of newly initiated leaves, suggesting that KN1 modulates GA activity in specific domains of the shoots (Bolduc and Hake 2009).

## Environmental influence on maize inflorescence architecture

Recent studies revealed how nutrient availability, redox homeostasis, and water use efficiency are essential for sustaining inflorescence development. The metalloid boron (B) is a structural components of plant cell walls and can serve as a cofactor in stabilizing protein structure or activating enzymes and may affect cellular activities such as signaling and membrane function. Two maize mutants, *tassel-less1* (*tls1*) and *rotten ear* (*rte*), provided molecular insights into the role of boron in maize inflorescence development. *TLS1* encodes an aquaporin protein that facilitates the movement of B and null mutations in *tls1* show defects in the early development of the SAM and IM when grown in boron-deficient conditions (Durbak et al. 2014). Similarly, null mutations of *rte* show defects in both vegetative and reproductive organs (Chatterjee et al. 2014). *RTE* encodes a membrane-localized boron efflux transporter, and it is expressed in cells surrounding the xylem, suggesting that RTE supplies adequate boron levels to developing inflorescences and that boron distribution in inflorescence meristems is essential for the formation of fertile reproductive organs (Chatterjee et al. 2014). Furthermore, the close paralog *RTE2* partially compensates *RTE* function during reproductive development and mutations in *rte2* severely enhance the defects of *rte* single mutants (Chatterjee et al. 2017). Overall, the function of *RTE*, *RTE2*, and *TLS1* are essential for shoot and root growth in soils with poor boron concentrations as well as for the formation of fully fertile inflorescences by maintaining meristem growth (Durbak et al. 2014). Boron deficiencies are common in certain areas of the world and lead to severe losses in different crop species, mainly because of reduced fertility. Understanding mechanisms for uptake and distribution of boron and other elements is therefore essential for devising strategies aimed at reducing losses in productivity in certain environments.

Redox homeostasis is attained by balancing the formation and removal of reactive oxygen species (ROS), and cells have evolved an effective defensive machinery for regulating excessive ROS levels. While ROS serve as essential signaling molecules for plants to respond to biotic and abiotic stresses, ROS crosstalk with hormonal pathways and meristem regulation is an active area of research (Zeng et al. 2017). Two recent studies have started to reveal the influence that ROS plays in maize inflorescence development. The maize *NEEDLE1* (*NDL1*) gene encodes a mitochondria-localized ATP-dependent metalloprotease involved in the stability and assembly of oxidative phosphorylation complexes, and null *ndl1* mutants strikingly resemble auxin-related mutants such as *vt2*, *spi1*, and *bif2* (Gallavotti et al. 2008; McSteen et al. 2007; Phillips et al. 2011). However, *ndl1* shows unique stress-associated defects and the accumulation of H_2_O_2_ in inflorescence meristems. This suggests that NDL1 malfunction in mitochondria increases ROS levels in inflorescence meristems that consequently alter endogenous auxin concentration, leading to defective initiation of suppressed bract primordia and axillary meristems in tassels and ears (Liu et al. 2019a).

Another gene involved in regulating meristem redox status was discovered by the characterization of the decussate phyllotaxy dominant mutant *Aberrant Phyllotaxis 2* (*Abph2*). *Abph2* is caused by a translocated copy of the *MSCA1* glutaredoxin (*GRX*) gene. Glutaredoxins are small ubiquitous redox enzymes that catalyze the reduction of disulfide bonds in target proteins (Fernandes and Holmgren 2004). As oxidation repair enzymes, glutaredoxins are involved in multiple cellular functions, such as redox signaling and control of glucose metabolism. Intriguingly, *Abph2* develops enlarged SAMs while the null *msca1*-*ref* mutant shows reduced SAM size. Moreover, protein-protein interaction assays revealed that MSCA1 interacts with the TGA-type bZIP transcription factor FEA4 (Pautler et al. 2015). FEA4 is an ortholog of Arabidopsis PERIANTHIA (PAN) that functions as a negative regulator of inflorescence meristem size independent of the CLV-WUS pathway (Pautler et al. 2015). All these results suggest that *MSCA1* positively regulates meristem size via regulation of FEA4 function, possibly by altering its redox status (Yang et al. 2015; Pautler et al. 2015).

In maize, water deficits and high temperatures during reproductive development and flowering result in widespread sterility, wilting and necrosis, and in a condition known as tassel blasting. Thus, these environmental factors have a major impact on inflorescence development and maize yield (Schoper et al. 1986; Schoper et al. 1987; McNellie et al. 2018; Dong et al. 2020; Begcy et al. 2019). However, how the availability of water and high temperatures affect pathways controlling maize inflorescence architecture is a much less studied topic, and the integration of environmental cues with plant meristem function is just beginning to be understood (Jones et al. 2021). Only a small number of candidate genes have been identified as maize drought resistance or heat tolerance genes via genome-wide association studies (GWAS). For example, a drought tolerance GWAS analysis in maize identified a MITE transposable element insertion in the promoter of a *NAC* gene (*ZmNAC111*) that is significantly associated with drought tolerance. Enhancing the expression of *ZmNAC111* by transgenic approaches led to increased drought tolerance in seedlings and increased water use efficiency (Mao et al. 2015). If and how these genes influence meristem development is currently unknown. Very recently, another NAC transcription factor, *NECROTIC UPPER TIPS1* (*NUT1*), encoding ZmNAC91, was shown to be required for long-distance water movement in maize plants by reinforcing the secondary cell wall in protoxylem cells and thus enabling these cells to withstand high negative pressures. Defects in *NUT1* function lead to visible necrosis and wilting of leaves, as well as sterility and blasting in tassels due to the decreased water supply (Dong et al. 2020). A deeper understanding of the molecular mechanisms regulating responses to water deficits and high temperatures and their interplay with inflorescence development is essential to reveal promising breeding targets and help maize tolerate the effects of rising global temperatures.

## Conclusions

As more and more genes and pathways are found to regulate the maintenance and activity of inflorescence meristems, new possibilities arise to engineer rational alterations of inflorescence architecture by exploiting existing natural variation in inbred lines, landraces, and even in the wild progenitor teosinte (Flint-Garcia 2013). Maize inbreds and landraces retain large nucleotide diversity (Hufford et al. 2012), and a large number of genes show presence/absence variations among inbreds (Lai et al. 2010). Therefore, there are abundant genetic resources potentially available to improve maize performance and improve yield outcomes in specific environments. While modern maize has been selected to have small upright tassels and large ears and to have an architecture adapted for high-density planting, this may not be the optimal solution for every environment. Tapping into diversity in landraces and teosinte may indeed yield a partial or complete reimagining of inflorescence architecture to match unique geographical and agricultural needs, including specific water and nutrient use strategies.

Furthermore, the ability to create targeted modifications in genes and regulatory regions by CRISPR-Cas-based approaches is poised to allow everexpanding possibilities to modify well-known pathways affecting inflorescence development and productivity and represents a promising area of translational research. In addition, the rapid selection and fixation of novel alleles in transgene-free offspring using these approaches dramatically shorten breeding programs (Eshed and Lippman 2019). Molecularly, we are just beginning to understand the transcriptional regulatory landscape of the maize genome, and recent efforts have provided valuable insights specific to inflorescence development (Galli et al. 2018; Ricci et al. 2019; Parvathaneni et al. 2020; Sun et al. 2020; Crisp et al. 2020). These efforts are expected to provide powerful information to guide practical applications in many crop species and provide a blueprint for maize genomic editing and new targets for maize improvement.

## Data Availability

Not applicable.

## References

[CR1] Bauer P, Lubkowitz M, Tyers R, Nemoto K, Meeley RB, Goff SA, Freeling M (2004). Regulation and a conserved intron sequence of liguleless3/4 knox class-I homeobox genes in grasses. Planta.

[CR2] Begcy K, Nosenko T, Zhou LZ, Fragner L, Weckwerth W, Dresselhaus T (2019). Male sterility in maize after transient heat stress during the tetrad stage of pollen development. Plant Physiol.

[CR3] Benkova E, Michniewicz M, Sauer M, Teichmann T, Seifertova D, Jurgens G, Friml J (2003). Local, efflux-dependent auxin gradients as a common module for plant organ formation. Cell.

[CR4] Bolduc N, Hake S (2009). The maize transcription factor KNOTTED1 directly regulates the gibberellin catabolism gene ga2ox1. Plant Cell.

[CR5] Bolduc N, Yilmaz A, Mejia-Guerra MK, Morohashi K, O’Connor D, Grotewold E, Hake S (2012). Unraveling the KNOTTED1 regulatory network in maize meristems. Genes Dev.

[CR6] Bolduc N, Tyers RG, Freeling M, Hake S (2014). Unequal redundancy in maize knotted1 homeobox genes. Plant Physiol.

[CR7] Bommert P, Lunde C, Nardmann J, Vollbrecht E, Running M, Jackson D, Hake S, Werr W (2005). Thick tassel dwarf1 encodes a putative maize ortholog of the Arabidopsis CLAVATA1 leucine-rich repeat receptor-like kinase. Development.

[CR8] Bommert P, Nagasawa NS, Jackson D (2013). Quantitative variation in maize kernel row number is controlled by the FASCIATED EAR2 locus. Nat Genet.

[CR9] Borras L, Vitantonio-Mazzini LN (2018). Maize reproductive development and kernel set under limited plant growth environments. J Exp Bot.

[CR10] Brand U, Fletcher JC, Hobe M, Meyerowitz EM, Simon R (2000). Dependence of stem cell fate in Arabidopsis on a feedback loop regulated by CLV3 activity. Science.

[CR11] Causier B, Ashworth M, Guo W, Davies B (2012). The TOPLESS interactome: a framework for gene repression in Arabidopsis. Plant Physiol.

[CR12] Chatterjee M, Tabi Z, Galli M, Malcomber S, Buck A, Muszynski M, Gallavotti A (2014). The boron efflux transporter ROTTEN EAR is required for maize inflorescence development and fertility. Plant Cell.

[CR13] Chatterjee M, Liu Q, Menello C, Galli M, Gallavotti A (2017). The combined action of duplicated boron transporters is required for maize growth in boron-deficient conditions. Genetics.

[CR14] Cheng PC, Greyson RI, Walden DB (1983). Organ initiation and the development of unisexual flowers in the tassel and ear of Zea mays. Am J Bot.

[CR15] Chuck GS, Brown PJ, Meeley R, Hake S (2014). Maize SBP-box transcription factors unbranched2 and unbranched3 affect yield traits by regulating the rate of lateral primordia initiation. Proc Natl Acad Sci U S A.

[CR16] Clark SE, Williams RW, Meyerowitz EM (1997). The CLAVATA1 gene encodes a putative receptor kinase that controls shoot and floral meristem size in Arabidopsis. Cell.

[CR17] Crisp PA, Marand AP, Noshay JM, Zhou P, Lu Z, Schmitz RJ, Springer NM (2020). Stable unmethylated DNA demarcates expressed genes and their cis-regulatory space in plant genomes. Proc Natl Acad Sci U S A.

[CR18] Doebley J (2004). The genetics of maize evolution. Annu Rev Genet.

[CR19] Dong Z, Xu Z, Xu L, Galli M, Gallavotti A, Dooner HK, Chuck G (2020). Necrotic upper tips1 mimics heat and drought stress and encodes a protoxylem-specific transcription factor in maize. Proc Natl Acad Sci U S A.

[CR20] Durbak AR, Phillips KA, Pike S, O’Neill MA, Mares J, Gallavotti A, Malcomber ST, Gassmann W, McSteen P (2014). Transport of boron by the tassel-less1 aquaporin is critical for vegetative and reproductive development in maize. Plant Cell.

[CR21] Eshed Y, Lippman ZB (2019). Revolutions in agriculture chart a coursresee for targeted breeding of old and new crops. Science.

[CR22] Eveland AL, Goldshmidt A, Pautler M, Morohashi K, Liseron-Monfils C, Lewis MW, Kumari S, Hiraga S, Yang F, Unger-Wallace E, Olson A, Hake S, Vollbrecht E, Grotewold E, Ware D, Jackson D (2014). Regulatory modules controlling maize inflorescence architecture. Genome Res.

[CR23] Fernandes AP, Holmgren A (2004). Glutaredoxins: glutathione-dependent redox enzymes with functions far beyond a simple thioredoxin backup system. Antioxid Redox Signal.

[CR24] Fischer KS, Edmeades GO, Johnson EC (1987). Recurrent selection for reduced tassel branch number and reduced leaf-area density above the ear in tropical maize populations. Crop Sci.

[CR25] Fletcher JC, Brand U, Running MP, Simon R, Meyerowitz EM (1999). Signaling of cell fate decisions by CLAVATA3 in Arabidopsis shoot meristems. Science.

[CR26] Flint-Garcia SA (2013). Genetics and consequences of crop domestication. J Agric Food Chem.

[CR27] Foster T, Yamaguchi J, Wong BC, Veit B, Hake S (1999). Gnarley1 is a dominant mutation in the knox4 homeobox gene affecting cell shape and identity. Plant Cell.

[CR28] Gaillochet C, Lohmann JU (2015). The never-ending story: from pluripotency to plant developmental plasticity. Development.

[CR29] Gallavotti A, Barazesh S, Malcomber S, Hall D, Jackson D, Schmidt RJ, McSteen P (2008). sparse inflorescence1 encodes a monocot-specific YUCCA-like gene required for vegetative and reproductive development in maize. Proc Natl Acad Sci U S A.

[CR30] Gallavotti A, Long JA, Stanfield S, Yang X, Jackson D, Vollbrecht E, Schmidt RJ (2010). The control of axillary meristem fate in the maize ramosa pathway. Development.

[CR31] Galli M, Liu Q, Moss BL, Malcomber S, Li W, Gaines C, Federici S, Roshkovan J, Meeley R, Nemhauser JL, Gallavotti A (2015). Auxin signaling modules regulate maize inflorescence architecture. Proc Natl Acad Sci U S A.

[CR32] Galli M, Khakhar A, Lu Z, Chen Z, Sen S, Joshi T, Nemhauser JL, Schmitz RJ, Gallavotti A (2018). The DNA binding landscape of the maize AUXIN RESPONSE FACTOR family. Nat Commun.

[CR33] Giulini A, Wang J, Jackson D (2004). Control of phyllotaxy by the cytokinin-inducible response regulator homologue ABPHYL1. Nature.

[CR34] Hake S, Smith HM, Holtan H, Magnani E, Mele G, Ramirez J (2004). The role of knox genes in plant development. Annu Rev Cell Dev Biol.

[CR35] Hay A, Tsiantis M (2010). KNOX genes: versatile regulators of plant development and diversity. Development.

[CR36] Huang P, Jiang H, Zhu C, Barry K, Jenkins J, Sandor L, Schmutz J, Box MS, Kellogg EA, Brutnell TP (2017). Sparse panicle1 is required for inflorescence development in Setaria viridis and maize. Nat Plants.

[CR37] Hufford MB, Xu X, van Heerwaarden J, Pyhajarvi T, Chia JM, Cartwright RA, Elshire RJ, Glaubitz JC, Guill KE, Kaeppler SM, Lai J, Morrell PL, Shannon LM, Song C, Springer NM, Swanson-Wagner RA, Tiffin P, Wang J, Zhang G, Doebley J, McMullen MD, Ware D, Buckler ES, Yang S, Ross-Ibarra J (2012). Comparative population genomics of maize domestication and improvement. Nat Genet.

[CR38] Hunter RB, Daynard TB, Hume DJ, Tanner JW, Curtis JD, Kannenberg LW (1969) Effect of tassel removal on grain yield of corn (Zea mays L). Crop Sci 9(4):405–406. 10.2135/cropsci1969.0011183X000900040003x1969.0011183X000900040003x

[CR39] Hussain HA, Men S, Hussain S, Chen Y, Ali S, Zhang S, Zhang K, Li Y, Xu Q, Liao C, Wang L (2019). Interactive effects of drought and heat stresses on morpho-physiological attributes, yield, nutrient uptake and oxidative status in maize hybrids. Sci Rep.

[CR40] Jackson D, Hake S (1999). Control of phyllotaxy in maize by the abphyl1 gene. Development.

[CR41] Je BI, Gruel J, Lee YK, Bommert P, Arevalo ED, Eveland AL, Wu Q, Goldshmidt A, Meeley R, Bartlett M, Komatsu M, Sakai H, Jonsson H, Jackson D (2016). Signaling from maize organ primordia via FASCIATED EAR3 regulates stem cell proliferation and yield traits. Nat Genet.

[CR42] Je BI, Xu F, Wu Q, Liu L, Meeley R, Gallagher JP, Corcilius L, Payne RJ, Bartlett ME, Jackson D (2018) The CLAVATA receptor FASCIATED EAR2 responds to distinct CLE peptides by signaling through two downstream effectors. Elife 7:e35673. 10.7554/eLife.35673 10.7554/eLife.35673 PMC5854466 29543153

[CR43] Jeong S, Trotochaud AE, Clark SE (1999). The Arabidopsis CLAVATA2 gene encodes a receptor-like protein required for the stability of the CLAVATA1 receptor-like kinase. Plant Cell.

[CR44] Jia H, Li M, Li W, Liu L, Jian Y, Yang Z, Shen X, Ning Q, Du Y, Zhao R, Jackson D, Yang X, Zhang Z (2020). A serine/threonine protein kinase encoding gene KERNEL NUMBER PER ROW6 regulates maize grain yield. Nat Commun.

[CR45] Jones DS, John A, VanDerMolen KR, Nimchuk ZL (2021). CLAVATA signaling ensures reproductive development in plants across thermal environments. Curr Biol.

[CR46] Kerstetter RA, Laudencia-Chingcuanco D, Smith LG, Hake S (1997). Loss-of-function mutations in the maize homeobox gene, knotted1, are defective in shoot meristem maintenance. Development.

[CR47] Kieffer M, Stern Y, Cook H, Clerici E, Maulbetsch C, Laux T, Davies B (2006). Analysis of the transcription factor WUSCHEL and its functional homologue in Antirrhinum reveals a potential mechanism for their roles in meristem maintenance. Plant Cell.

[CR48] Kitagawa M, Jackson D (2019). Control of meristem size. Annu Rev Plant Biol.

[CR49] Knauer S, Javelle M, Li L, Li X, Ma X, Wimalanathan K, Kumari S, Johnston R, Leiboff S, Meeley R, Schnable PS, Ware D, Lawrence-Dill C, Yu J, Muehlbauer GJ, Scanlon MJ, Timmermans MCP (2019). A high-resolution gene expression atlas links dedicated meristem genes to key architectural traits. Genome Res.

[CR50] Krogan NT, Hogan K, Long JA (2012). APETALA2 negatively regulates multiple floral organ identity genes in Arabidopsis by recruiting the co-repressor TOPLESS and the histone deacetylase HDA19. Development.

[CR51] Lai J, Li R, Xu X, Jin W, Xu M, Zhao H, Xiang Z, Song W, Ying K, Zhang M, Jiao Y, Ni P, Zhang J, Li D, Guo X, Ye K, Jian M, Wang B, Zheng H, Liang H, Zhang X, Wang S, Chen S, Li J, Fu Y, Springer NM, Yang H, Wang J, Dai J, Schnable PS, Wang J (2010). Genome-wide patterns of genetic variation among elite maize inbred lines. Nat Genet.

[CR52] Li M, Zhong W, Yang F, Zhang Z (2018). Genetic and molecular mechanisms of quantitative trait loci controlling maize inflorescence architecture. Plant Cell Physiol.

[CR53] Liu L, Du Y, Shen X, Li M, Sun W, Huang J, Liu Z, Tao Y, Zheng Y, Yan J, Zhang Z (2015). KRN4 controls quantitative variation in maize kernel row number. PLoS Genet.

[CR54] Liu Q, Galli M, Liu X, Federici S, Buck A, Cody J, Labra M, Gallavotti A (2019). NEEDLE1 encodes a mitochondria localized ATP-dependent metalloprotease required for thermotolerant maize growth. Proc Natl Acad Sci U S A.

[CR55] Liu X, Galli M, Camehl I, Gallavotti A (2019). RAMOSA1 ENHANCER LOCUS2-mediated transcriptional repression regulates vegetative and reproductive architecture. Plant Physiol.

[CR56] Long JA, Ohno C, Smith ZR, Meyerowitz EM (2006). TOPLESS regulates apical embryonic fate in Arabidopsis. Science.

[CR57] Lu Z, Shao G, Xiong J, Jiao Y, Wang J, Liu G, Meng X, Liang Y, Xiong G, Wang Y, Li J (2015). MONOCULM 3, an ortholog of WUSCHEL in rice, is required for tiller bud formation. J Genet Genomics.

[CR58] Mao H, Wang H, Liu S, Li Z, Yang X, Yan J, Li J, Tran LS, Qin F (2015). A transposable element in a NAC gene is associated with drought tolerance in maize seedlings. Nat Commun.

[CR59] Mayer KF, Schoof H, Haecker A, Lenhard M, Jurgens G, Laux T (1998). Role of WUSCHEL in regulating stem cell fate in the Arabidopsis shoot meristem. Cell.

[CR60] McNellie JP, Chen JP, Li XR, Yu JM (2018). Genetic mapping of foliar and tassel heat stress tolerance in maize. Crop Sci.

[CR61] McSteen P, Malcomber S, Skirpan A, Lunde C, Wu X, Kellogg E, Hake S (2007). Barren inflorescence2 encodes a co-ortholog of the PINOID serine/threonine kinase and is required for organogenesis during inflorescence and vegetative development in maize. Plant Physiol.

[CR62] Meng WJ, Cheng ZJ, Sang YL, Zhang MM, Rong XF, Wang ZW, Tang YY, Zhang XS (2017). Type-B ARABIDOPSIS RESPONSE REGULATORs specify the shoot stem cell niche by dual regulation of WUSCHEL. Plant Cell.

[CR63] Michniewicz M, Brewer PB, Friml JI (2007). Polar auxin transport and asymmetric auxin distribution. Arabidopsis Book.

[CR64] Moss BL, Mao H, Guseman JM, Hinds TR, Hellmuth A, Kovenock M, Noorassa A, Lanctot A, Villalobos LI, Zheng N, Nemhauser JL (2015). Rate motifs tune auxin/indole-3-acetic acid degradation dynamics. Plant Physiol.

[CR65] Muehlbauer GJ, Fowler JE, Girard L, Tyers R, Harper L, Freeling M (1999). Ectopic expression of the maize homeobox gene liguleless3 alters cell fates in the leaf. Plant Physiol.

[CR66] Muszynski MG, Moss-Taylor L, Chudalayandi S, Cahill J, Del Valle-Echevarria AR, Alvarez-Castro I, Petefish A, Sakakibara H, Krivosheev DM, Lomin SN, Romanov GA, Thamotharan S, Dam T, Li B, Brugiere N (2020). The maize Hairy Sheath Frayed1 (Hsf1) mutation alters leaf patterning through increased cytokinin signaling. Plant Cell.

[CR67] Nardmann J, Werr W (2006). The shoot stem cell niche in angiosperms: expression patterns of WUS orthologues in rice and maize imply major modifications in the course of mono- and dicot evolution. Mol Biol Evol.

[CR68] Parvathaneni RK, Bertolini E, Shamimuzzaman M, Vera DL, Lung PY, Rice BR, Zhang J, Brown PJ, Lipka AE, Bass HW, Eveland AL (2020). The regulatory landscape of early maize inflorescence development. Genome Biol.

[CR69] Pautler M, Eveland AL, LaRue T, Yang F, Weeks R, Lunde C, Je BI, Meeley R, Komatsu M, Vollbrecht E, Sakai H, Jackson D (2015). FASCIATED EAR4 encodes a bZIP transcription factor that regulates shoot meristem size in maize. Plant Cell.

[CR70] Phillips KA, Skirpan AL, Liu X, Christensen A, Slewinski TL, Hudson C, Barazesh S, Cohen JD, Malcomber S, McSteen P (2011). Vanishing tassel2 encodes a grass-specific tryptophan aminotransferase required for vegetative and reproductive development in maize. Plant Cell.

[CR71] Ramos Baez R, Buckley Y, Yu H, Chen Z, Gallavotti A, Nemhauser JL, Moss BL (2020). A synthetic approach allows rapid characterization of the maize nuclear auxin response circuit. Plant Physiol.

[CR72] Ricci WA, Lu Z, Ji L, Marand AP, Ethridge CL, Murphy NG, Noshay JM, Galli M, Mejia-Guerra MK, Colome-Tatche M, Johannes F, Rowley MJ, Corces VG, Zhai J, Scanlon MJ, Buckler ES, Gallavotti A, Springer NM, Schmitz RJ, Zhang X (2019). Widespread long-range cis-regulatory elements in the maize genome. Nat Plants.

[CR73] Rodriguez-Leal D, Lemmon ZH, Man J, Bartlett ME, Lippman ZB (2017). Engineering quantitative trait variation for crop improvement by genome editing. Cell.

[CR74] Rodriguez-Leal D, Xu C, Kwon CT, Soyars C, Demesa-Arevalo E, Man J, Liu L, Lemmon ZH, Jones DS, Van Eck J, Jackson DP, Bartlett ME, Nimchuk ZL, Lippman ZB (2019). Evolution of buffering in a genetic circuit controlling plant stem cell proliferation. Nat Genet.

[CR75] Schneeberger RG, Becraft PW, Hake S, Freeling M (1995). Ectopic expression of the knox homeo box gene rough sheath1 alters cell fate in the maize leaf. Genes Dev.

[CR76] Schoof H, Lenhard M, Haecker A, Mayer KF, Jurgens G, Laux T (2000). The stem cell population of Arabidopsis shoot meristems in maintained by a regulatory loop between the CLAVATA and WUSCHEL genes. Cell.

[CR77] Schoper JB, Lambert RJ, Vasilas BL (1986). Maize pollen viability and ear receptivity under water and high-temperature stress. Crop Sci.

[CR78] Schoper JB, Lambert RJ, Vasilas BL, Westgate ME (1987). Plant factors controlling seed set in maize : the influence of silk, pollen, and ear-leaf water status and tassel heat treatment at pollination. Plant Physiol.

[CR79] Schwechheimer C, Willige BC (2009). Shedding light on gibberellic acid signalling. Curr Opin Plant Biol.

[CR80] Serrano-Mislata A, Bencivenga S, Bush M, Schiessl K, Boden S, Sablowski R (2017). DELLA genes restrict inflorescence meristem function independently of plant height. Nat Plants.

[CR81] Shi B, Guo X, Wang Y, Xiong Y, Wang J, Hayashi KI, Lei J, Zhang L, Jiao Y (2018). Feedback from lateral organs controls shoot apical meristem growth by modulating auxin transport. Dev Cell.

[CR82] Skirpan A, Culler AH, Gallavotti A, Jackson D, Cohen JD, McSteen P (2009). BARREN INFLORESCENCE2 interaction with ZmPIN1a suggests a role in auxin transport during maize inflorescence development. Plant Cell Physiol.

[CR83] Somssich M, Je BI, Simon R, Jackson D (2016). CLAVATA-WUSCHEL signaling in the shoot meristem. Development.

[CR84] Sun Y, Dong L, Zhang Y, Lin D, Xu W, Ke C, Han L, Deng L, Li G, Jackson D, Li X, Yang F (2020). 3D genome architecture coordinates trans and cis regulation of differentially expressed ear and tassel genes in maize. Genome Biol.

[CR85] Taguchi-Shiobara F, Yuan Z, Hake S, Jackson D (2001). The fasciated ear2 gene encodes a leucine-rich repeat receptor-like protein that regulates shoot meristem proliferation in maize. Genes Dev.

[CR86] Tanaka W, Hirano HY (2020). Antagonistic action of TILLERS ABSENT1 and FLORAL ORGAN NUMBER2 regulates stem cell maintenance during axillary meristem development in rice. New Phytol.

[CR87] Tanaka W, Ohmori Y, Ushijima T, Matsusaka H, Matsushita T, Kumamaru T, Kawano S, Hirano HY (2015). Axillary meristem formation in rice requires the WUSCHEL ortholog TILLERS ABSENT1. Plant Cell.

[CR88] Trung KH, Tran QH, Bui NH, Tran TT, Luu KQ, Tran NTT, Nguyen LT, Nguyen DTN, Vu BD, Quan DTT, Nguyen DT, Nguyen HT, Dang CC, Tran BM, Khanh TD, Vi SL (2020). A weak allele of FASCIATED EAR 2 (FEA2) increases maize kernel row number (KRN) and yield in elite maize hybrids. Agronomy-Basel.

[CR89] Tsuda K, Abraham-Juarez MJ, Maeno A, Dong Z, Aromdee D, Meeley R, Shiroishi T, Nonomura KI, Hake S (2017). KNOTTED1 cofactors, BLH12 and BLH14, regulate internode patterning and vein anastomosis in maize. Plant Cell.

[CR90] Vollbrecht E, Schmidt RJ (2009) Development of the inflorescences. In: Handbook of maize: its biology, vol I. Springer-Verlag New York, pp 13-40. 10.1007/978-0-387-79418-1

[CR91] Vollbrecht E, Reiser L, Hake S (2000). Shoot meristem size is dependent on inbred background and presence of the maize homeobox gene, knotted1. Development.

[CR92] Vollbrecht E, Springer PS, Goh L, Buckler ES, Martienssen R (2005). Architecture of floral branch systems in maize and related grasses. Nature.

[CR93] Wang L, Kim J, Somers DE (2013). Transcriptional corepressor TOPLESS complexes with pseudoresponse regulator proteins and histone deacetylases to regulate circadian transcription. Proc Natl Acad Sci U S A.

[CR94] Wang Q, Kohlen W, Rossmann S, Vernoux T, Theres K (2014). Auxin depletion from the leaf axil conditions competence for axillary meristem formation in Arabidopsis and tomato. Plant Cell.

[CR95] Wang Y, Wang J, Shi B, Yu T, Qi J, Meyerowitz EM, Jiao Y (2014). The stem cell niche in leaf axils is established by auxin and cytokinin in Arabidopsis. Plant Cell.

[CR96] Wang J, Tian C, Zhang C, Shi B, Cao X, Zhang TQ, Zhao Z, Wang JW, Jiao Y (2017). Cytokinin signaling activates WUSCHEL expression during axillary meristem initiation. Plant Cell.

[CR97] Weijers D, Wagner D (2016). Transcriptional responses to the auxin hormone. Annu Rev Plant Biol.

[CR98] Westgate ME, Lizaso J, Batchelor W (2003). Quantitative relationships between pollen shed density and grain yield in maize. Crop Sci.

[CR99] Wu Q, Xu F, Liu L, Char SN, Ding Y, Je BI, Schmelz E, Yang B, Jackson D (2020). The maize heterotrimeric G protein beta subunit controls shoot meristem development and immune responses. Proc Natl Acad Sci U S A.

[CR100] Xie M, Chen H, Huang L, O’Neil RC, Shokhirev MN, Ecker JR (2018). A B-ARR-mediated cytokinin transcriptional network directs hormone cross-regulation and shoot development. Nat Commun.

[CR101] Xu G, Wang X, Huang C, Xu D, Li D, Tian J, Chen Q, Wang C, Liang Y, Wu Y, Yang X, Tian F (2017). Complex genetic architecture underlies maize tassel domestication. New Phytol.

[CR102] Yadav RK, Perales M, Gruel J, Girke T, Jonsson H, Reddy GV (2011). WUSCHEL protein movement mediates stem cell homeostasis in the Arabidopsis shoot apex. Genes Dev.

[CR103] Yang F, Bui HT, Pautler M, Llaca V, Johnston R, Lee BH, Kolbe A, Sakai H, Jackson D (2015). A maize glutaredoxin gene, abphyl2, regulates shoot meristem size and phyllotaxy. Plant Cell.

[CR104] Yao H, Skirpan A, Wardell B, Matthes MS, Best NB, McCubbin T, Durbak A, Smith T, Malcomber S, McSteen P (2019). The barren stalk2 gene is required for axillary meristem development in maize. Mol Plant.

[CR105] Zeng J, Dong Z, Wu H, Tian Z, Zhao Z (2017). Redox regulation of plant stem cell fate. EMBO J.

[CR106] Zhang F, Wang Y, Li G, Tang Y, Kramer EM, Tadege M (2014). STENOFOLIA recruits TOPLESS to repress ASYMMETRIC LEAVES2 at the leaf margin and promote leaf blade outgrowth in Medicago truncatula. Plant Cell.

[CR107] Zhang D, Sun W, Singh R, Zheng Y, Cao Z, Li M, Lunde C, Hake S, Zhang Z (2018). GRF-interacting factor1 regulates shoot architecture and meristem determinacy in maize. Plant Cell.

[CR108] Zhou Y, Liu X, Engstrom EM, Nimchuk ZL, Pruneda-Paz JL, Tarr PT, Yan A, Kay SA, Meyerowitz EM (2015). Control of plant stem cell function by conserved interacting transcriptional regulators. Nature.

[CR109] Zhou Y, Yan A, Han H, Li T, Geng Y, Liu X, Meyerowitz EM (2018). HAIRY MERISTEM with WUSCHEL confines CLAVATA3 expression to the outer apical meristem layers. Science.

[CR110] Zubo YO, Blakley IC, Yamburenko MV, Worthen JM, Street IH, Franco-Zorrilla JM, Zhang W, Hill K, Raines T, Solano R, Kieber JJ, Loraine AE, Schaller GE (2017). Cytokinin induces genome-wide binding of the type-B response regulator ARR10 to regulate growth and development in Arabidopsis. Proc Natl Acad Sci U S A.

